# Supplementary material for: Condensation of Rubisco into a proto-pyrenoid in higher plant chloroplasts
Source: Nat Commun. 2020 Dec 9;11:6303. doi: 10.1038/s41467-020-20132-0 (PMC7726157; doi:10.1038/s41467-020-20132-0)
Supplement: Supplementary file 1 — Supplementary Information [file 41467_2020_20132_MOESM1_ESM.pdf]

# Condensation of Rubisco into a proto-pyrenoid in higher plant chloroplasts

Atkinson *et al.*

**Supplementary Table 1. Photosynthetic parameters from gas exchange and fluorescence measurements for $S2_{Cr}$ transgenic lines of Arabidopsis.**

| | Ep1 | Az1 | Ep2 | Az2 | Ep3 | Az3 | EpWt | AzWt |
|---|---|---|---|---|---|---|---|---|
| $V_{cmax}$ (µmol $CO_2$ $m^{-2}$ $s^{-1}$) | 35.6±1.5[a] | 36.4±2.0[a] | 32.2±1.9[a] | 33.6±1.6[a] | 33.1±1.9[a] | 33.8±2.2[a] | 44.9±1.6[b] | 43.3±1.7[b] |
| $J_{max}$ (µmol $e^-$ $m^{-2}$ $s^{-1}$) | 59.2±2.3[a] | 61.9±6.3[a] | 57.2±2.6[a] | 56.1±3.5[a] | 52.9±4.4[a] | 58.6±5.2[a] | 76.4±2.4[b] | 74.9±7.5[b] |
| Γ (µmol $CO_2$ $m^{-2}$ $s^{-1}$) | 63±8[a] | 53±5[a] | 52±6[a] | 54±7[a] | 53±7[a] | 56±8[a] | 51±7[a] | 64±12[a] |
| $g_s$ (mol $H_2O$ $m^{-2}$ $s^{-1}$) | 0.249±0.031[a] | 0.279±0.051[a] | 0.233±0.017[a] | 0.251±0.015[a] | 0.233±0.021[a] | 0.236±0.016[a] | 0.287±0.018[a] | 0.306±0.011[a] |
| $g_m$ (mol $CO_2$ $m^{-2}$ $s^{-1}$) | 0.034±0.001[b] | 0.035±0.003[b] | 0.032±0.002[b] | 0.033±0.002[b] | 0.034±0.003[b] | 0.032±0.002[b] | 0.045±0.002[a] | 0.046±0.003[a] |
| $F_v/F_m$ (ML) | 0.848±0.002[a] | 0.849±0.002[a] | 0.848±0.001[a] | 0.847±0.001[a] | 0.847±0.002[a] | 0.845±0.002[a] | 0.851±0.002[a] | 0.850±0.001[a] |
| $F_v/F_m$ (HL) | 0.852±0.002[a] | 0.845±0.002[a] | 0.850±0.001[a] | 0.855±0.004[a] | 0.846±0.002[a] | 0.849±0.001[a] | 0.850±0.003[a] | 0.852±0.002[a] |

Note: The mean and SEM are shown for 35- to 45-d-old rosettes for gas exchange variables ($n$ = 5-8), and 32-d-old rosettes for $F_v/F_m$. $F_v/F_m$ is shown for attached leaves dark-adapted for 45 min prior to fluorescence measurements ($n$ = 6-23). Letters above the SEM indicate significant difference ($p < 0.05$) as determined by one-way ANOVA followed by Tukey's HSD tests. Values followed by the same letter are not statistically significantly different. Abbreviations: Γ, $CO_2$ compensation point ($C_i$–$A$); $A$, $CO_2$ assimilation rate, $C_i$, inorganic carbon concentration; $F_v/F_m$, maximum potential quantum efficiency of photosystem II; $g_s$, stomatal conductance to water vapour; $g_m$, mesophyll conductance to $CO_2$ (i.e. conductance of $CO_2$ across the pathway from intercellular airspace to chloroplast stroma) using the Ethier and Livingston method (see Methods section); $J_{max}$, maximum electron transport rate; $V_{cmax}$, maximum rate of Rubisco carboxylation. Source data are provided as a Source Data file.

**Supplementary Table 2. Transcript abundances of tGFP and eGFP in EPYC1-dGFP plant lines.**

| | tGFP expression | | | eGFP expression | | |
|---|---|---|---|---|---|---|
| | Rep 1 | Rep 2 | Average | Rep 1 | Rep 2 | Average |
| Ep1 | 0.222 | 0.294 | 0.258 | 0.299 | 0.315 | 0.307 |
| Ep2 | 0.857 | 0.482 | 0.669 | 1.093 | 0.524 | 0.808 |
| Ep3 | 1.430 | 0.569 | 1 | 1.7E-05 | 4.98E-05 | 3.34E-05 |

Note: Quantitative reverse transcription PCR (RT-qPCR) was carried out on three T2 $S2_{Cr}$ transgenic plants expressing EPYC1-dGFP (Ep1 - Ep3, as in Fig. 1B) using gene-specific primers for tGFP and eGFP. Abundances of tGFP and eGFP transcripts are shown relative to the highest tGFP expression level and normalised to reference genes PP2A (At1g13320) and RHIP1 (At4g26410). Each biological replicate shown (e.g. Rep 1) is an average of two technical replicates.

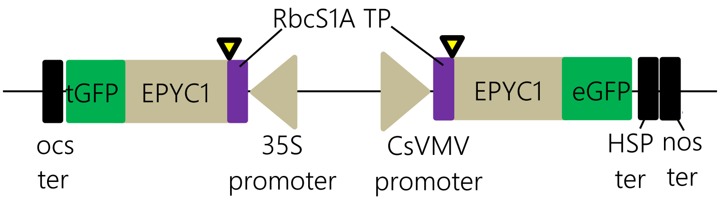


```
ocs terminator
turbo GFP (Pontellina pluma)
mature EPYC1
Rbcs1A transit peptide
35S promoter
linking region
CSVMV promoter
eGFP (with N-terminal HA-tag)
HSP terminator
nos terminator
```

```
GTCCTGCTGAGCCTCGACATGTTGTCGCAAAATTCGCCCTGGACCCGCCCAACGATTTGTCGTCACTGTCAAGGT
TTGACCTGCACTTCATTTGGGGCCCACATACACCAAAAAAATGCTGCATAATTCTCGGGGCAGCAAGTCGGTTAC
CCGGCCGCCGTGCTGGACCGGGTTGAATGGTGCCCGTAACTTTCGGTAGAGCGGACGGCCAATACTCAACTTCAA
GGAATCTCACCCATGCGCGCCGGCGGGGAACCGGAGTTCCCTTCAGTGAGCGTTATTAGTTCGCCGCTCGGTGTG
TCGTAGATACTAGCCCCTGGGGCACTTTTGAAATTTGAATAAGATTTATGTAATCAGTCTTTTAGGTTTGACCGG
TTCTGCCGCTTTTTTTAAAATTGGATTTGTAATAATAAAACGCAATTGTTTGTTATTGTGGCGCTCTATCATAGA
TGTCGCTATAAACCTATTCAGCACAATATATTGTTTTCATTTTAATATTGTACATATAAGTAGTAGGGTACAATC
AGTAAATTGAACGGAGAATATTATTCATAAAAATACGATAGTAACGGGTGATATATTCATTAGAATGAACCGAAA
CCGGCGGTAAGGATCTGAGCTACACATGCTCAGGTTTTTTACAACGTGCACAACAGAATTGAAAGCAAATATCAT
GCGATCATAGGCTTCTCGCATATCTCATTAAAGCAGGACaagcTTATCATTCCTCACCAGCATCAGCATCAGGGG
TCTTGAAAGCATGTTGGTACTCAACGATTCCAAGCTCGGTGTTAGAGTGATCTTCCTCAACTCTTCTGAAAGCGA
ACATAGGTCCACCGTTTTGAAGGATAGAAGGGTGGATAGCAGACTTGAAGTGCATGTGAGAATCCACCACAGAAG
AGTAGTAACCACCATCTCTAAGTGAGAAGGTTCTGGTGAAAGATCCATCGAGATCGTTATCTCCCATAGGATGAA
GATGCTCAACAGTAGCGTTAGACCTGATGATCTTGTCGGTGAAGATAACAGAATCCTCAGGGAATCCAGTTCCCA
TAACCTGCACATCAACAAATTTTGGTCATATATTAGAAAAGTTATAAATTAAAATATACACACTTATAAACTACA
GAAAAGCAATTGCTATATACTACATTCTTTTATTTTGAAAAAAATATTTGAAATATTATATTACTACTAATTAAT
GATAATTATTATATATATATATCAAAGGTAGAAGCAGAAACTTACCTTGAAATCTCCGATCACTCTTCCAGCCTCGT
ATCTGTAAGAGAAGCTAACGTGAAGAACACCACCATCCTCGTACTTCTCGATCCTAGTGTTGGTGTATCCACCGT
TGTTGATAGCATGAAGGAAAGGGTTCTCGTATCCAGATGGGTAAGTTCCGAAGTGGTAGAATCCGTATCCCATAA
CGTGAGAAAGAAGGTATGGAGAGAAGGTAAGAGCACCCTTGGTAGACTTCATCTTGTTAGTCATTCTTCCCTGCT
CAGGAGTTCCCTCACCACCTCCAACAAGCTCGAACTCAACACCGTTAAGGGTTCCAGTGATTCTACACTCGATTT
CCATAGCAGGAAGTCCAGACTCATCAGACTCAGATCCAGATCCTCTcgaaAGGCCCTTTCTCCAGTCTGCAGGGA
GAGCCGTTCTCTTAATTTCTGGCTTGCTTTTACCTGTCCAAGGATTAGTTCCGGCTTTGTCAGCGGAGGAACTAG
ATGACGAAGCGGGAGCGTCTCTCCAGGAAGCGGATGATGATCTAGCAGGTGCAGGGGCACTGCTTGCAGGTGCGG
GACTGTTGGATCGGAGTGACTCCAACTCCTGTTTCCAATTACTTGGAAGGGCCGATCTACTAGGGGTGACTGCTT
TTTTGCTCGCCGATGAGCTACGAGCTGGGGCGGAAGAAGCAGGAGCTGCATCCCTCCAACTAGCTGAGGATGACC
TAGCTGGTGCTGGCGCGCTAGAAGCTGGTGCTGGACTGCTTGATCGAAGGGATTCAAGTTCCTGTTTCCAGTTAG
AAGGCAGAGCTGAACGAGACGGAGTGACGGCCTTCTTGCTGGAAGAACTCCTAGCTGGAGCGCTAGATGCAGGCG
CTGCGTCCCTCCATGAGGCAGAACTACTTCTGGCCGGAGCTGGCGCTGATGAAGCCGGTGCAGGGCTGGAGCTTC
TCAATGACTCCAATTCCTGCTTCCAATTTGAAGGCAAGGCACTTCTTGAAGGAGTAACGGCCTTCTTTGAAGCTG
AGCTACTTCTGGCAGGCGCTGATGATGCAGGAGCGGCGTCTCGCCACGATGCGCTGCTCGATCTCGCAGGAGCAG
GAGCAGAGGACGCCGCCGACGAGCTGCCGTTACCATTTCTGAGTGATTCAAGTTCTTGCCTCCAATTAGCTGGGA
GAACGCTTCTTGTTGGGCTCACTCTGTTCGTTGCACTACTAGCTCTTGAGGCTTGAACTGTAGCAGTAGATGACT
CTCTCCAAGATCCTCTTGCTGCacctCCGCAGTTAACTCTTCCGCCGTTGCTTGTGATGGAAGTAATGTCGTTGT
TAGCCTTGCGGGTGGCTGGGAAGGCAGCGGAGGACTTAAGTCCGTTGAAAGGAGCGACCATAGTGGCCTGAGCCG
GAGAGGCAACCATAGTAGCGGAAGAGAGCATAGAGGAAGCcattGTATCGATAATTGTAAATGTAATTGTAATGT
TGTTTGTTGTTTGTTGTTGTTGGTAATTGTTGTAAAAATGAGCTCTTATACTCGAGCGTGTCCTCTCCAAATGAA
```

ATGAACTTCCTTATATAGAGGAAGGGTCTTGCGAAGGATAGTGGGATTGTGCGTCATCCCTTACGTCAGTGGAGA
TGTCACATCAATCCACTTGCTTTGTAGACGTGGTTGGAACCTCTTCTTTTTCCACGATGCTCCTCGTGGGTGGGG
GTCCATCTTTGGGACCACTGTCGGCAGAGAGATCTTGAATGATAGCCTTTCCTTTATCGCAATGATGGCATTTGT
AGGAGCCACCTTCCTTTTCTACTGTCCTTTCGATGAAGTGACAGATAGCTGGGCAATGGAATCCGAGGAGGTTTC
CCGAAATTATCCTTTGTTGAAAAGTCTCAATAGCCCTTTGATCTTCTGAGACTGTATCTTTGACATTTTTGGAGT
AGACCAGAGTGTCGTGCTCCACCATGTTGACcctcGCAAGAATTCAAGCTTggagCCAGAAGGTAATTATCCAAG
ATGTAGCATCAAGAATCCAATGTTTACGGGAAAAACTATGGAAGTATTATGTAAGCTCAGCAAGAAGCAGATCAA
TATGCGGCACATATGCAACCTATGTTCAAAAATGAAGAATGTACAGATACAAGATCCTATACTGCCAGAATACGA
AGAAGAATACGTAGAAATTGAAAAAGAAGAACCAGGCGAAGAAAAGAATCTTGATGACGTAAGCACTGACGACAA
CAATGAAAAGAAGAAGATAAGGTCGGTGATTGTGAAAGAGACATAGAGGACACATGTAAGGTGGAAAATGTAAGG
GCGGAAAGTAACCTTATCACAAAGGAATCTTATCCCCCACTACTTATCCTTTTATATTTTTCCGTGTCATTTTTG
CCCTTGAGTTTTCCTATATAAGGAACCAAGTTCGGCATTTGTGAAAACAAGAAAAAATTTGGTGTAAGCTATTTT
CTTTGAAGTACTGAGGATACAACTTCAGAGAAATTTGTAAGTTTGTaatgGCTTCCTCTATGCTCTCTTCCGCTA
CTATGGTTGCCTCTCCGGCTCAGGCCACTATGGTCGCTCCTTTCAACGGACTTAAGTCCTCCGCTGCCTTCCCAG
CCACCCGCAAGGCTAACAACGACATTACTTCCATCACAAGCAACGGCGGAAGAGTTAACTGCGGaggtGCAGCAA
GAGGATCTTGGAGAGAGTCATCTACTGCTACAGTTCAAGCCTCAAGAGCTAGTAGTGCAACGAACAGAGTGAGCC
CAACAAGAAGCGTTCTCCCAGCTAATTGGAGGCAAGAACTTGAATCACTCAGAAATGGTAACGGCAGCTCGTCGG
CGGCGTCCTCTGCTCCTGCTCCTGCGAGATCGAGCAGCGCATCGTGGCGAGACGCCGCTCCTGCATCATCAGCGC
CTGCCAGAAGTAGCTCAGCTTCAAAGAAGGCCGTTACTCCTTCAAGAAGTGCCTTGCCTTCAAATTGGAAGCAGG
AATTGGAGTCATTGAGAAGCTCCAGCCCTGCACCGGCTTCATCAGCGCCAGCTCCGGCCAGAAGTAGTTCTGCCT
CATGGAGGGACGCAGCGCCTGCATCTAGCGCTCCAGCTAGGAGTTCTTCCAGCAAGAAGGCCGTCACTCCGTCTC
GTTCAGCTCTGCCTTCTAACTGGAAACAGGAACTTGAATCCCTTCGATCAAGCAGTCCAGCACCAGCTTCTAGCG
CGCCAGCACCAGCTAGGTCATCCTCAGCTAGTTGGAGGGATGCAGCTCCTGCTTCTTCCGCCCCAGCTCGTAGCT
CATCGGCGAGCAAAAAAGCAGTCACCCCTAGTAGATCGGCCCTTCCAAGTAATTGGAAACAGGAGTTGGAGTCAC
TCCGATCCAACAGTCCCGCACCTGCAAGCAGTGCCCCTGCACCTGCTAGATCATCATCCGCTTCCTGGAGAGACG
CTCCCGCTTCGTCATCTAGTTCCTCCGCTGACAAAGCCGGAACTAATCCTTGGACAGGTAAAAGCAAGCCAGAAA
TTAAGAGAACGGCTCTCCCTGCAGACTGGAGAAAGGGCCTTtcgTACCCATACGATGTTCCTGACTATGCGGGCT
ATCCCTATGACGTCCCGGACTATGCAGGATTGTATCCATATGACGTTCCAGATTACGCCACTAGAGCTGCTTACC
CATACGATGTTCCTGACTATGCGGGCTATCCCTATGACGTCCCGGACTATGCAGGATTGTATCCATATGACGTTC
CAGATTACGCCGTGAGCAAGGGCGAGGAGCTGTTCACCGGGGTGGTGCCCATCCTGGTCGAGCTGGACGGCGACG
TAAACGGCCACAAGTTCAGCGTGTCCGGCGAGGGCGAGGGCGATGCCACCTACGGCAAGCTGACCCTGAAGTTCA
TCTGCACCACCGGCAAGCTGCCCGTGCCCTGGCCCACCCTCGTGACCACCCTGACCTACGGCGTGCAGTGCTTCA
GCCGCTACCCCGACCACATGAAGCAGCACGACTTCTTCAAGTCCGCCATGCCCGAAGGCTACGTCCAGGAGCGCA
CCATCTTCTTCAAGGACGACGGCAACTACAAGACCCGCGCCGAGGTGAAGTTCGAGGGCGACACCCTGGTGAACC
GCATCGAGCTGAAGGGCATCGACTTCAAGGAGGACGGCAACATCCTGGGGCACAAGCTGGAGTACAACTACAACA
GCCACAACGTCTATATCATGGCCGACAAGCAGAAGAACGGCATCAAGGTGAACTTCAAGATCCGCCACAACATCG
AGGACGGCAGCGTGCAGCTCGCCGACCACTACCAGCAGAACACCCCCATCGGCGACGGCCCCGTGCTGCTGCCCG
ACAACCACTACCTGAGCACCCAGTCCGCCCTGAGCAAAGACCCCAACGAGAAGCGCGATCACATGGTCCTGCTGG
AGTTCGTGACCGCCGCCGGGATCACTCTCGGCATGGACGAGCTGTACAAGTAAgcttATATGAAGATGAAGATGA
AATATTTGGTGTGTCAAATAAAAAGCTTGTGTGCTTAAGTTTGTGTTTTTTTCTTGGCTTGTTGTGTTATGAATT
TGTGGCTTTTTCTAATATTAAATGAATGTAAGATCTCATTATAATGAATAAACAAATGTTTCTATAATCCATTGT
GAATGTTTTGTTGGATCTCTTCTGCAGCATATAACTACTGTATGTGCTATGGTATGGACTATGGAATATGATTAA
AGATAAGagatGTCAAGCAGATCGTTCAAACATTTGGCAATAAAGTTTCTTAAGATTGAATCCTGTTGCCGGTCT
TGCGATGATTATCATATAATTTCTGTTGAATTACGTTAAGCATGTAATAATTAACATGTAATGCATGACGTTATT
TATGAGATGGGTTTTTATGATTAGAGTCCCGCAATTATACATTTAATACGCGATAGAAAACAAAATATAGCGCGC
AAACTAGGATAAATTATCGCGCGCGGTGTCATCTATGTTACTAGATCGA

**Supplementary Figure 1. Full sequence of the EPYC1 expression cassettes.** Sequence parts are colour coded, and MoClo overhangs are shown in lower case. Source data are provided as a Source Data file.

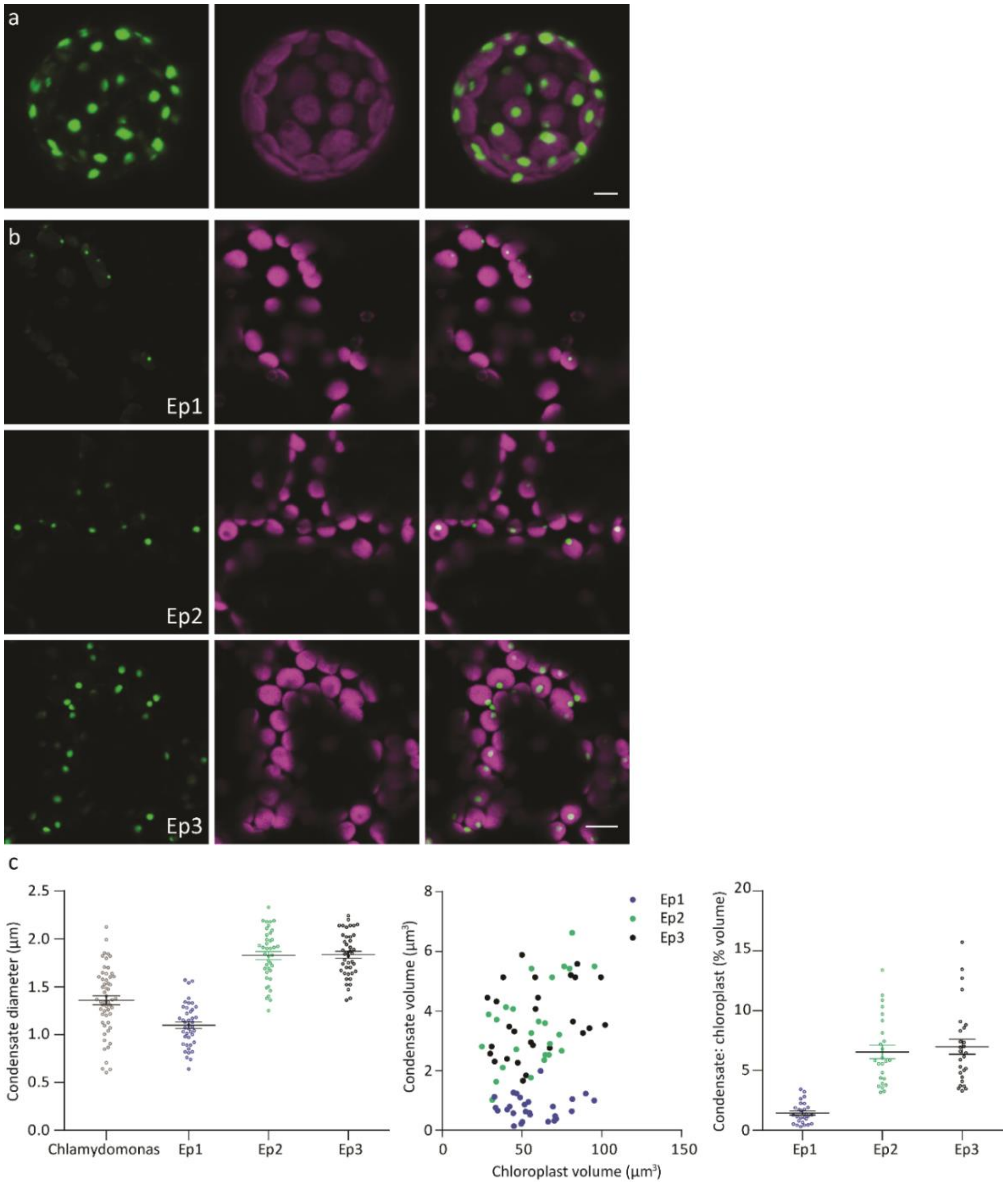


**Supplementary Figure 2. Characterisation of condensates in S2$_{Cr}$ plants. a**, Maximum projection of a z stack showing condensates in every chloroplast. Scale bar = 5 µm for all images. Similar stacked images were reproduced for six individual protoplasts. **b**, The size of condensates is dependent on the expression level of EPYC1. Arabidopsis lines Ep1-3 with different expression levels of EPYC1-dGFP (see Figure. 1b) have different sizes of condensates. Scale bar = 10 µm for all images. Confocal images are representative of images captured from Ep1 plants (42), Ep2 plants (21) and Ep3 plants (>100). **c**, Data derived from confocal images of Chlamydomonas pyrenoids (*n* = 55) and chloroplasts (*n* = 40-42) from each of the three Ep transgenic lines (Ep1-3) showing the mean diameter and SEM of pyrenoids and condensates (left). The volume of the condensate is shown plotted against estimated chloroplast volume (centre) and the estimated proportion of chloroplast volume occupied by the condensate (right) (*n* = 25-27 chloroplasts for each line). Source data underlying Supplementary Figure 2c are provided as a Source Data file. Raw image data corresponding to Supplementary Figure 2c are available online at the open access Edinburgh DataShare repository (https://doi.org/10.7488/ds/2945).

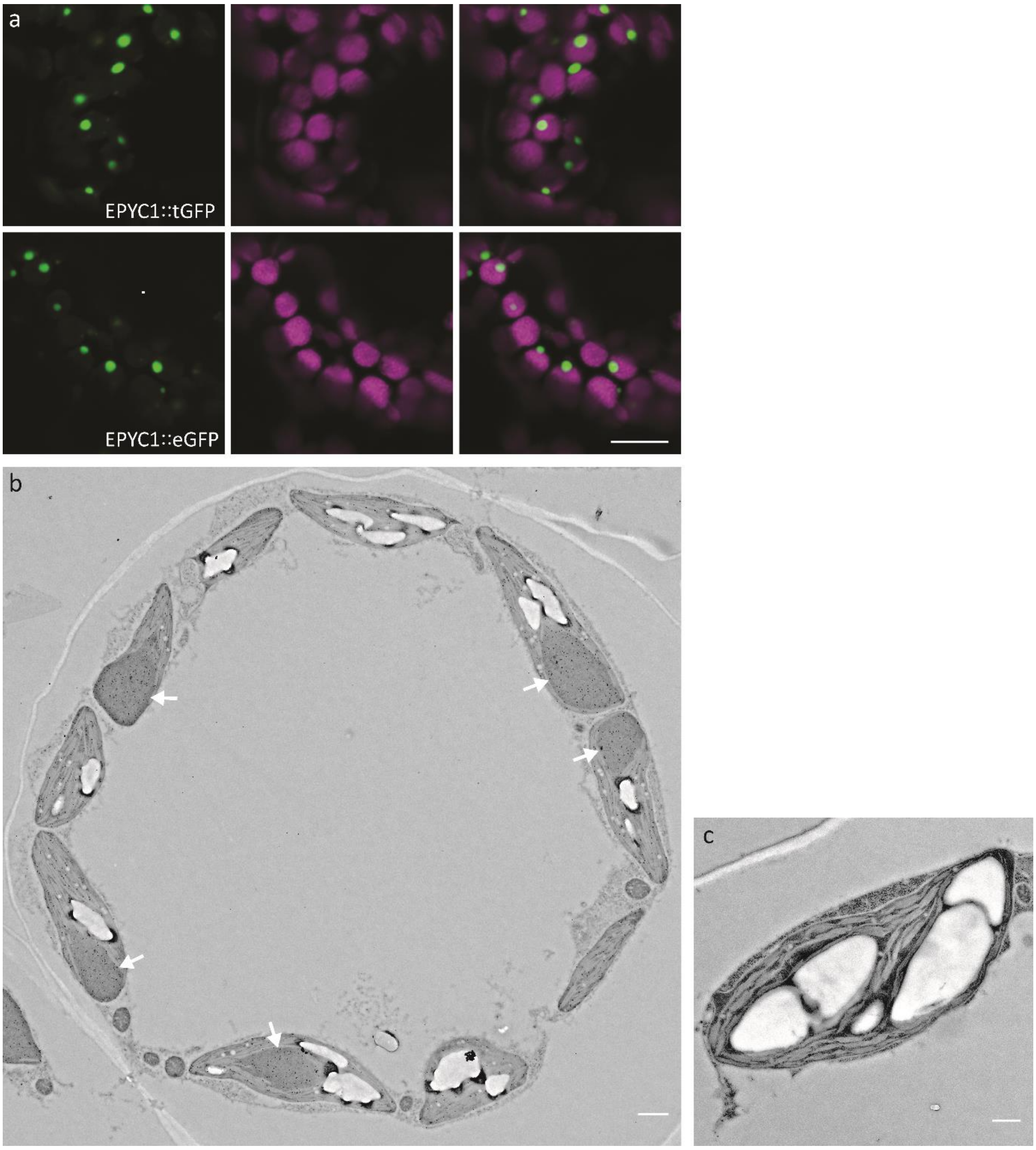


**Supplementary Figure 3. Condensate formation in S2$_{Cr}$ plants. a**, Condensation occurs in S2$_{Cr}$ plants expressing a single EPYC1 expression cassette. Representative images are shown for EPYC1 fused at the C-terminus to either tGFP (top) or eGFP (bottom). Scale bar = 10 µm. Confocal images are representative of images captured from EPYC1:tGFP plants (14) and EPYC1:tGFP plants (17). **b**, TEM image of a mesophyll cell cross-section showing chloroplasts with EPYC1-dGFP condensates. Visible condensates are marked by a white arrowhead. The section has been probed by immunogold labelling with anti-Rubisco antibodies. Scale bar = 1 µm. The TEM image is representative of 5 images. c, TEM image of a chloroplast from a wild-type plant expressing EPYC1-dGFP. No condensates were observed in this background. Scale bar = 0.5 µm. The TEM image is representative of 5 images.

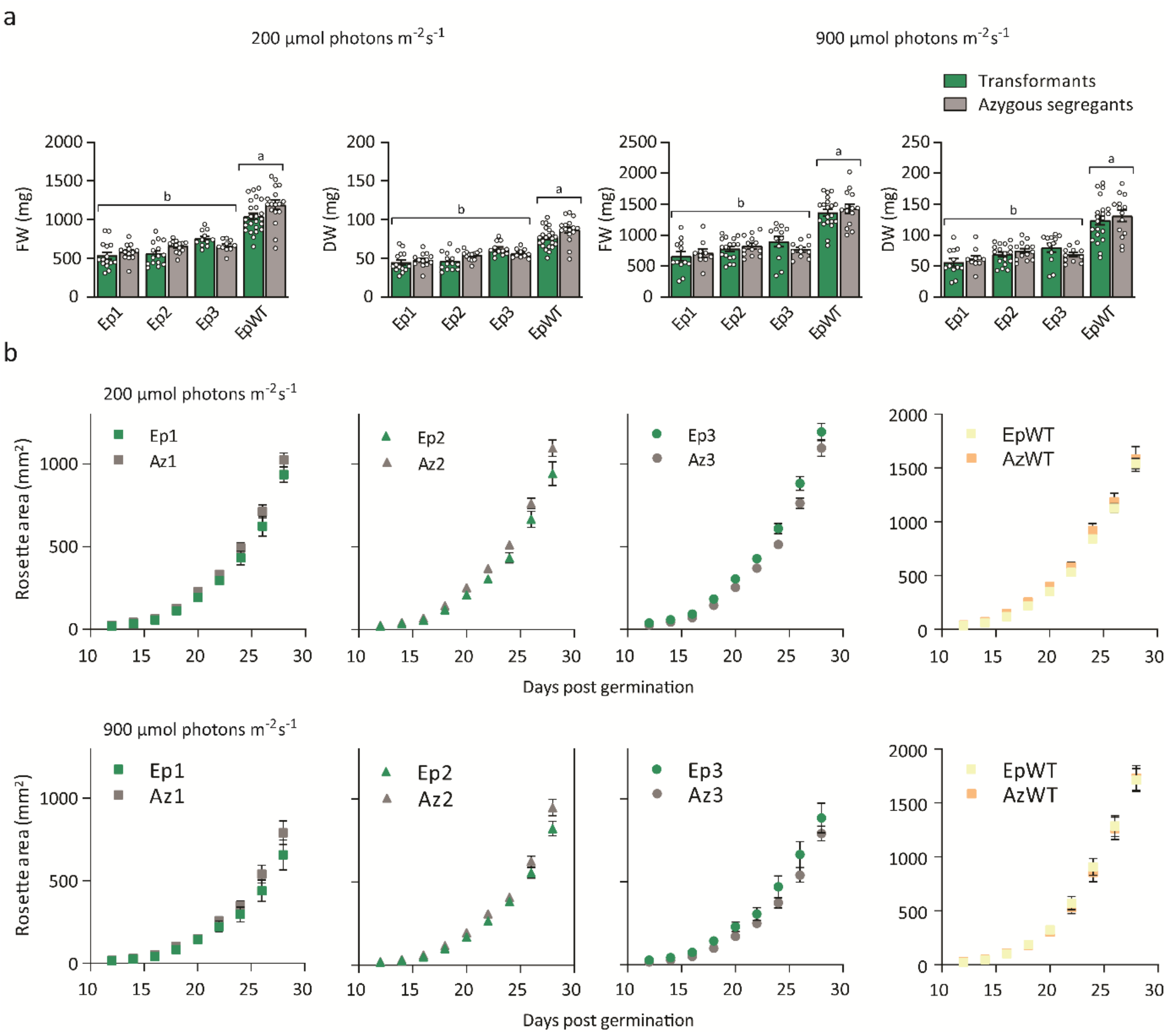


**Supplementary Figure 4. Growth of plant lines expressing EPYC1 under different light levels. a,** Fresh (FW) and dry weight (DW) of three T2 EPYC1-dGFP $S2_{Cr}$ transgenic lines (Ep1-3, green) and an EPYC1-dGFP WT transformant (EpWT, yellow) with their respective azygous segregants (Az1-3, grey and AzWT, orange) measured after 32 days of growth at 200 or 900 µmol photons $m^{-2}$ $s^{-1}$ light. The mean ± SEM are shown for n=10-26 individual plants for each line. Letters indicate significant difference ($p < 0.05$) of EpWT lines compared to Ep lines as determined by one-way ANOVA followed by Tukey's honestly significant difference (HSD) post-hoc tests. **b,** Rosette area expansion rates for individual transgenic lines and azygous segregants at two different light levels in (a). Source data are provided as a Source Data file.
