# Supplementary material for: Condensation of Rubisco into a proto-pyrenoid in higher plant chloroplasts
Source: Nat Commun. 2020 Dec 9;11:6303. doi: 10.1038/s41467-020-20132-0 (PMC7726157; doi:10.1038/s41467-020-20132-0)
Supplement: Supplementary file 3 — Reporting Summary [file 41467_2020_20132_MOESM3_ESM.pdf]

nature research

Corresponding author(s): Alistair McCormick

Last updated by author(s): Nov 6, 2020

Reporting Summary

Nature Research wishes to improve the reproducibility of the work that we publish. This form provides structure for consistency and transparency in reporting. For further information on Nature Research policies, see our Editorial Policies and the Editorial Policy Checklist.

Statistics

For all statistical analyses, confirm that the following items are present in the figure legend, table legend, main text, or Methods section.

n/a	Confirmed	
☐	☒	The exact sample size (*n*) for each experimental group/condition, given as a discrete number and unit of measurement
☐	☒	A statement on whether measurements were taken from distinct samples or whether the same sample was measured repeatedly
☐	☒	The statistical test(s) used AND whether they are one- or two-sided<br>*Only common tests should be described solely by name; describe more complex techniques in the Methods section.*
☐	☒	A description of all covariates tested
☒	☐	A description of any assumptions or corrections, such as tests of normality and adjustment for multiple comparisons
☐	☒	A full description of the statistical parameters including central tendency (e.g. means) or other basic estimates (e.g. regression coefficient) AND variation (e.g. standard deviation) or associated estimates of uncertainty (e.g. confidence intervals)
☐	☒	For null hypothesis testing, the test statistic (e.g. F, t, r) with confidence intervals, effect sizes, degrees of freedom and P value noted<br>*Give P values as exact values whenever suitable.*
☒	☐	For Bayesian analysis, information on the choice of priors and Markov chain Monte Carlo settings
☒	☐	For hierarchical and complex designs, identification of the appropriate level for tests and full reporting of outcomes
☒	☐	Estimates of effect sizes (e.g. Cohen's d, Pearson's r), indicating how they were calculated

Our web collection on statistics for biologists contains articles on many of the points above.

Software and code

Policy information about availability of computer code

Data collection	Data collection described in Methods section. No custom software was used.
Data analysis	Fiji (ImageJ, v1.52n) and Image Studio Lite (LICOR v5.2) were used for western blot band analyses. Fiji (ImageJ, v1.52n) was used for TEM image analyses. Fiji (ImageJ, v1.52n) and Leica software were used confocal image analyses. Microsoft Word and Excel (2016) were used to capture data and GraphPad Prism (v8) was used for generating graphs. Statistical analyses was performed in IBM SPSS Statistics (v26). Figures were generated in Adobe Illustrator CS3.

For manuscripts utilizing custom algorithms or software that are central to the research but not yet described in published literature, software must be made available to editors and reviewers. We strongly encourage code deposition in a community repository (e.g. GitHub). See the Nature Research guidelines for submitting code & software for further information.

Data

Policy information about availability of data

All manuscripts must include a data availability statement. This statement should provide the following information, where applicable:

- Accession codes, unique identifiers, or web links for publicly available datasets
- A list of figures that have associated raw data
- A description of any restrictions on data availability

The datasets and plant materials generated and analysed during the current study are available from the corresponding author upon request. A reporting summary for this article is available as a Supplementary Information file. Data supporting the findings of this work are available within the paper and its Supplementary Information files. The source data underlying Figures 1-4, Supplementary Table 1 and Supplementary Figures 1, 2c, and 4 are provided as a Source Data file. Raw image data corresponding to Figure 1, Figure 3e and Supplementary Figure 2c are available online on Edinburgh DataShare (https://doi.org/10.7488/ds/2945).

Field-specific reporting

Please select the one below that is the best fit for your research. If you are not sure, read the appropriate sections before making your selection.

☒ Life sciences ☐ Behavioural & social sciences ☐ Ecological, evolutionary & environmental sciences

For a reference copy of the document with all sections, see nature.com/documents/nr-reporting-summary-flat.pdf

Life sciences study design

All studies must disclose on these points even when the disclosure is negative.

Sample size	Sample size was determined based on standards for experimental plant biology studies, attempting to have a minimum of n = 5 biological replicates with sufficient reproducibility.
Data exclusions	No data was excluded.
Replication	No findings were not replicable. Statistical analysis was conducted on a minimum five individual biological samples for each experiment. Replication of experimental data is also indicated in the manuscript.
Randomization	Samples within lines were selected randomly for image analysis. Plant samples were randomised within the growth incubator during growth experiments and subsequent gas exchange analyses.
Blinding	Data collection and analysis was not performed blind.

Reporting for specific materials, systems and methods

We require information from authors about some types of materials, experimental systems and methods used in many studies. Here, indicate whether each material, system or method listed is relevant to your study. If you are not sure if a list item applies to your research, read the appropriate section before selecting a response.

Materials & experimental systems

n/a	Involved in the study	
☐	☒	Antibodies
☒	☐	Eukaryotic cell lines
☒	☐	Palaeontology and archaeology
☒	☐	Animals and other organisms
☒	☐	Human research participants
☒	☐	Clinical data
☒	☐	Dual use research of concern

Methods

n/a	Involved in the study	
☒	☐	ChIP-seq
☒	☐	Flow cytometry
☒	☐	MRI-based neuroimaging

Antibodies

Antibodies used	Polyclonal wheat anti-Rubisco primary antibody (Howe et al., 1983), CrRbcS2 primary antibody (Eurogentec, Southampton, UK), actin primary antibody (66009-1-Ig, Proteintech, UK), IRDye 800CW goat anti-rabbit IgG secondary antibody (LI-COR Biotechnology, Cambridge, UK).
Validation	The Rubisco primary antibody has been used in several publication since being produced in 1983. It has no lot number, serum was shared with us several years ago from the Griffith's Lab (Cambridge, UK). The CrRbcS2 primary antibody was raised and validation performed by Eurogentec. A validation statement is provided by Proteintech on the manufacturer's website for the Actin primary antibody, and by LI-COR Biotechnology for the IRDye 800CW goat anti-rabbit IgG secondary antibody.
