# Supplementary material for: Condensation of Rubisco into a proto-pyrenoid in higher plant chloroplasts
Source: Nat Commun. 2020 Dec 9;11:6303. doi: 10.1038/s41467-020-20132-0 (PMC7726157; doi:10.1038/s41467-020-20132-0)
Supplement: Supplementary file 4 — Description of Additional Supplementary Files [file 41467_2020_20132_MOESM4_ESM.pdf]

Description of Additional Supplementary Files

Supplementary Movie 1
Reconstruction of SIM image data showing the EPYC1-dGFP 25 condensates inside chloroplasts in Arabidopsis.
