# Supplementary material for: Condensation of Rubisco into a proto-pyrenoid in higher plant chloroplasts
Source: Nat Commun. 2020 Dec 9;11:6303. doi: 10.1038/s41467-020-20132-0 (PMC7726157; doi:10.1038/s41467-020-20132-0)
Supplement: Supplementary file 2 — Peer Review File [file 41467_2020_20132_MOESM2_ESM.pdf]

Reviewer #1 (Remarks to the Author):

Increasing the efficiency of photosynthetic CO2 fixation in C3 plants is an important and challenging task for meeting the increasing world food demand. One possible strategy chosen by the authors of this manuscript is to reconstitute the well-known algal CO2 concentrating mechanism (CCM) in C3 plants. A key component of this system is the pyrenoid, a liquid-like condensate of Rubisco and EPYC1 present in algae. The authors showed previously that hybrid Arabidopsis Rubisco containing the Chlamydomonas SSU could phase separate into liquid droplets with purified EPYC1 in vitro,. In this manuscript the authors have created transgenic Arabidopsis lines expressing EPYC1 and a hybrid plant-algal Rubisco and shown that this pyrenoid-like aggregation of Rubisco also occurs in vivo. They also show that the photosynthetic CO2 assimilation and growth are not affected compared to control plants. This work represents an important advance but, as the authors recognize, several other problems will have to be solved for rebuilding a functional CCM system in crop plants.

Minor remarks
l.59 replace truncated by mature EPYC1
L93 Contrary to what is stated the levels of EPYC1 in the EPYC1::tGFP or EPYC1::eGFP lines are comparatively higher than with EPYC1-dGFP (Fig. 1b).

Reviewer #2 (Remarks to the Author):

The authors have presented a very cohesive and exciting piece of work that has provided an important first step to install a pyrenoid into higher plant chloroplasts. The authors induced liquid-liquid phase separation (LLPS) of Arabidopsis Rubisco harbouring Chlamydmonas SSu's or Modified Arabidopsis SSu's (containing Chlamy residues to interact with EPYC1) into a liquid-like condensate by correctly expressing the mature form of EPYC1. The liquid-like condensate is similar to that already characterised in Chlamydomonas by the Jonikas group. The complete installation of the Chlammy pyrenoid into plants is a significant challenge due the lack of success of inserting bicarbonate transporters into chloroplast membranes (either the plasma membrane or thylakoid membrane) that are appropriately energised and the lack of understanding in the thylakoid invaginations that are important for functional pyrenoids in Chlamydomonas. These events coupled with correct localization of Carbonic anhydrase are required to significantly raise the CO2 concentration around Rubisco to suppress oxygenation. Nevertheless, this is a significant advance that will appeal across many disciplines. Furthermore, the modifications required to induce the proto-pyrenoid were achievable through nucleus transformation of EPYC1 and Chlamydomonas SSu or a modified Rubisco SSu, which may open opportunities to explore the installation of pyrenoids in monocot crops which aren't amenable to plastid-transformation. The authors demonstrated further that the transgenic Arabidopsis plants were photosynthetically competent with CO2 assimilation mirroring azygous plants. This suggests so far that CO2 is able to penetrate the proto-pyrenoid. Overall, CO2 assimilation in transgenic plants was lower than WT Arabidopsis because of the changes to Rubisco kinetics and content as a result of the Chlammy SSU/ Arabidopsis LSu hybrid Rubisco enzyme.
Overall, this is an outstanding piece of work and the authors have nicely designed and executed the experiments with the appropriate controls. I have a number of points for the authors to consider:
1) Line 15 – It is best to add here that the pyrenoid CCM in Chlammy is inducible under conditions of low inorganic carbon present in the surrounding liquid environment.
2) Line 17 – The authors should define the acronym EPYC1 here and cite the original PNAS paper for its finding.
3) Lines 54-56: The authors mention a truncated form. I think chimeric form is better terminology. Or just the mature peptide of EPYC1. However, it is more like the mature EPYC1 peptide fused to the Arabidopsis RbcS1A transit peptide is required for proper chloroplast import within Arabidopsis and the subsequent removal of the TP by the stromal peptidase. Is there evidence from recent work by the authors that cleavage of the native EPYC1 TP from the full length EPYC1 peptide was occurring plants?

Or is it a target of plant proteases because of incorrect processing or failure to assemble with Rubisco? Can the authors determine if the RbcS1A TP is correctly cleaved?
4) The authors prudently used two different versions of GFP fused to EPYC1. It appears that tGFP is responsible for elevated expression or the promoter is more efficient. The promoters should be defined in the legend in Figure 1 and also in the methods. Secondly, is the codon usage identical for both GFP coding sequences in each fusion? It is quite interesting as to why EPYC fused to tGFP is more expressed and/or assembles more readily with Rubisco. Is the mRNA for the EPYC::tGFP construct more abundant? The lack of degradation products implies that EPYC1, which is structurally disordered, is protected from proteases once assembled with Rubisco.
5) The authors haven't cited the review by Wunder et al 2019 – Traffic. This is relevant to the study and describes appropriate background to EPYC1 and LLPS.
6) The authors mention that the size of the condensate is likely related to expression level of EPYC1 but there is still degradation intermediates. Presumably, the free Rubisco could form another condensate within the chloroplast or is there some molecular crowding influence as well that governs this process? The authors do provide evidence it's not chloroplast size which further suggests the microenvironment is somehow limiting the assembly.
7) The authors mention that 52% of Arabidopsis Rubisco is incorporated into the proto-pyrenoid. This make sense since figure 3b shows a 1:1 ratio of Cr SSu's to Arabidopsis SSu's. However in Line 125, do the authors mean that in the condensate 81% of the SSu's are from Chlammy? This sentence is not clear. From figure 3b it appears that there is no Arabidopsis SSu's assembled in the condensate. However, the western blot shows perhaps a small amount. Perhaps a longer exposure maybe required for the western blot to accurately determine this. However, if there are no Arabidopsis SSu's present in the condensate, this also suggests that the SSu's are not assembled as mixed populations and points toward assembly is limited to one SSu population only. This needs to be further clarified.
8) The photosynthetic competency of transgenic plants are similar to azygous controls which suggests that CO_2 can penetrate the proto-pyrenoid to Rubisco molecules close the central part of the condensate. This is further evidenced by similarities in Vcmax. Have the authors measured Rubisco activation status? This might be important because within the condensate Rubisco activase might not be able to penetrate to remove inhibitors from the active sites of Rubisco. Because CO_2 is not elevated within the proto-pyrenoid (as there is no bicarbonate pumps), it is likely that a significant proportion of Rubisco is not activated or competent for catalysis.
9) It is great to see that the authors have used the appropriate Arabidopsis kinetic parameters to model the ACi curves to determine Vcmax and other parameters. However, I am not sure if the method described by authors to determine mesophyll conductance is robust enough. Generally, it is accepted that the most accurate method for determining gm is online carbon isotope discrimination coupled to photosynthesis measurements using the Licor-6400.
10) As the authors point out, alterations to the Rubisco LSu requires plastid transformation that is not available in key C3 monocot crops, however, work by Salesse-Smith et al., 2018 Nature Plants, strongly suggests that there is an ability to express Rubisco LSu from the nucleus in maize plants. The authors should be aware of this work.
11) Lines 149-151 – The authors suggest here that decreases in Rubisco content is the cause of differences in CO_2 assimilation between Transgenic plants and WT. The authors have recently published the kinetic parameters of the hybrid Arabidopsis LSU / Chlammy SSu Rubisco which were significantly different from the WT Arabidopsis. The >10% reduction in the carboxylation efficiency of the hybrid Rubisco and decreases in Kcat and Sc/o is sufficient to induce changes in CO_2 assimilation. A lower Rubisco content X a lower kcat reduces in vitro Vcmax significantly. I would suggest the authors reword this text to include reductions in Rubisco content and changes in Rubisco kinetics are responsible for the changes in CO_2 assimilation.

Reviewer #3 (Remarks to the Author):

The manuscript by Atkinson et al. reports on an approach to introduce the algal CO2 concentrating mechanism (CCM, a functional Rubisco condensate) into plant chloroplasts. In the chloroplasts of eukaryotic algae, Rubisco together with the linker protein EYPC1 localizes into a phase-separated compartment called pyrenoid. High concentrations of CO2 in the pyrenoid enhance the carboxylation function of Rubisco. Such a microcompartment is absent in C3 plants and the Rubisco operates under sub-optimal CO2 conditions. To increase crop yields, one important strategy would be to introduce an algal pyrenoid-like or cyanobacterial carboxysome-like Rubisco condensate into higher plant chloroplasts. Here the authors report the first successful attempt of forming a single phase separated compartment in Arabidopsis chloroplasts upon expression of EYPC1 and a plant-algal hybrid Rubisco. Although the Rubisco/EYPC1 condensate displaced the thylakoid membranes, the authors observed no impairment in Rubisco function and only a mild impairment in plant growth.
The approach reported by Atkinson et al. is promising, requiring modification of the nuclear-encoded SSU and possibly introducing a minimal set of genes to create an efficient algal CCM in plants. This strategy is possibly easier than introducing the CCM of cyanobacteria, which requires many more genes for carboxysome formation in plants.

Major points:
1. The authors used S2Cr instead of 1AAtMOD to carry out the whole study. 1AAtMOD has a better turnover rate and specificity comparing with S2Cr. Furthermore, as reported previously, the amount of total Rubisco is 75% of wild-type levels for 1AAtMOD, and 65% for S2Cr. Can the authors comment why they did not use the 1AAtMOD strain for this initial critical study of making a proto-pyrenoid in higher plant chloroplasts.
2. Lines 63-65: The statement that EPYC1::tGFP is generally more highly expressed than EPYC1::eGFP is not supported by the data shown in Figure 1b, the loading actin control for EPYC1::eGFP is much lower than that for EPYC1::tGFP.
3. Lines 92-93: Again the statement "However, the overall levels of EPYC1 expression in these lines were comparatively lower (Fig. 1b)." is not supported by the data shown in Figure 1b. In Fig. 1b, the EPYC1::eGFP or EPYC1::tGFP strains expressing "eGFP" and "tGFP" show higher levels of the respective proteins than in Ep1, Ep2 or Ep3.
4. Description of the FRAP and chemical cross-linking experiments are missing in Methods section.
5. Please specify which transgenic Ep plant (1, 2 or 3) was used in the respective figures.
6. In Figs. 3a and 3b the amount of protein in the input is lower than the combined intensity of supernatant and pellet. Please clarify. The present figure is misleading.
7. It is surprising that essentially no AtRbcS was detected in the pelleted condensate (Figure 3b). It is hard to imagine that all eight RbcS binding sites on Rubisco are occupied exclusively by CrRbcS, considering that the total amount of AtRbcS protein appears to be slightly higher than CrRbcS2 (Figure 3b).
8. The values for Vmax and Jmax are not consistent with those reported previously in Atkinson, N. et al. (2017). Can the authors explain the difference?

Minor points:
1. Figure 1d; show also TEM of WT cells. Please indicate the "dense body" by an arrow (or arrowhead) and explain/describe what the white large structures are in the legend.
2. Figure 1f: why is Rubisco condensate not seen in all chloroplast organelles?
3. Figure 1f: please describe the magenta puncta in the figure legend.
4. Figures 1f and 1g: What are the light pink/white structures in the condensate? Are these regions representing overlap of the condensate and thylakoid chlorophyll?
5. Please keep the images within the same figure panel in the same scale. This applies to Figure 1c, Extended Data Figure 2b and Extended Data Figure 3a.
6. Figure 2a: A dotted line at y=1.0 would be helpful to indicate that the fluorescence signal has been normalized to 1.
7. In Figure 2b the position of bleach point should have been in the middle of the condensate to better visualize recovery. The pre-bleach image of the fixed sample looks overexposed. Was the display

setting for both samples the same? Was the fluorescence of GFP lower after fixing? Could this be due to denaturation of GFP?

8. Lines 119 & 123, & Figure 3b: Correct spelling ‘Coomassie’

9. Figure 3d: In the image of CrRbcS2, what are the large dark circular clusters? The authors should indicate the chloroplasts with a dotted outline and indicate the Rubisco condensate. Again what are the large white structures?

10. Figure 3e is not described or mentioned in the text. Why is the y-axis scale set at a maximum of 1.5 when the maximum value of data is only 1.0?

11. Extended Data Figure 3b: please indicate the chloroplasts with observable Rubisco condensates and also the scale bar is missing.

12. A better description of Az lines would be helpful for a non-expert.

13. Please site Figure 1c and the previous publication at the end of the statement (lines 153-156), “Condensate formation in the 1AAtMOD background, where catalytic characteristics of the hybrid Rubisco are indistinguishable from that of WT Rubisco, indicates that the SSU can be further engineered to optimise phase separation, and Rubisco content and performance.”

14. Please site Figure 4d at the end of the statement (lines 145-147), “Notably, the CO2 assimilation rates at ambient concentrations of CO2 for Ep and Az lines were comparable to WT lines when normalized for Rubisco content (ARubisco)”.

15. Extended Data Figure 4 legend, change “a)” and “b)” to “a,” and “b,”, respectively.

RESPONSE TO REVIEWER COMMENTS

Reviewer #1 (Remarks to the Author):

Increasing the efficiency of photosynthetic CO2 fixation in C3 plants is an important and challenging task for meeting the increasing world food demand. One possible strategy chosen by the authors of this manuscript is to reconstitute the well-known algal CO2 concentrating mechanism (CCM) in C3 plants. A key component of this system is the pyrenoid, a liquid-like condensate of Rubisco and EPYC1 present in algae. The authors showed previously that hybrid Arabidopsis Rubisco containing the Chlamydomonas SSU could phase separate into liquid droplets with purified EPYC1 in vitro,. In this manuscript the authors have created transgenic Arabidopsis lines expressing EPYC1 and a hybrid plant-algal Rubisco and shown that this pyrenoid-like aggregation of Rubisco also occurs in vivo. They also show that the photosynthetic CO2 assimilation and growth are not affected compared to control plants. This work represents an important advance but, as the authors recognize, several other
problems will have to be solved for rebuilding a functional CCM system in crop plants.

Minor remarks
l.59 replace truncated by mature EPYC1
- We have removed 'truncated' from lines 56, 61 and 205, and added 'mature' to line 61.

L93 Contrary to what is stated the levels of EPYC1 in the EPYC1::tGFP or EPYC1::eGFP lines are comparatively higher than with EPYC1-dGFP (Fig. 1b).
- We agree and have removed this sentence (now on line 97).

Reviewer #2 (Remarks to the Author):
The authors have presented a very cohesive and exciting piece of work that has provided an important first step to install a pyrenoid into higher plant chloroplasts. The authors induced liquid-liquid phase separation (LLPS) of Arabidopsis Rubisco harbouring Chlamydmonas SSu's or Modified Arabidopsis SSu's (containing Chlamy residues to interact with EPYC1) into a liquid-like condensate by correctly expressing the mature form of EPYC1. The liquid-like condensate is similar to that already characterised in Chlamydomonas by the Jonikas group. The complete installation of the Chlammy pyrenoid into plants is a significant challenge due the lack of success of inserting bicarbonate transporters into chloroplast membranes (either the plasma membrane or thylakoid membrane) that are appropriately energised and the lack of understanding in the thylakoid invaginations that are important for functional pyrenoids in Chlamydomonas. These events coupled with correct localization of Carbonic anhydrase are required to significantly raise the CO2 concentration around Rubisco to suppress oxygenation. Nevertheless, this is a significant advance that will appeal across many disciplines. Furthermore, the modifications required to induce the proto-pyrenoid were achievable through nucleus transformation of EPYC1 and Chlamydomonas SSu or a modified Rubisco SSu, which may open opportunities to explore the installation of pyrenoids in monocot crops which aren't amenable to plastid-transformation. The authors demonstrated further that the transgenic Arabidopsis plants were photosynthetically competent with CO2 assimilation mirroring azygous plants. This suggests so far that CO2 is able to penetrate the proto-pyrenoid. Overall, CO2 assimilation in transgenic plants was lower than WT Arabidopsis because of the changes to Rubisco kinetics and content as a result of the Chlammy SSU/ Arabidopsis LSu hybrid Rubisco enzyme.
Overall, this is an outstanding piece of work and the authors have nicely designed and executed the experiments with the appropriate controls. I have a number of points for the authors to consider:
1) Line 15 – It is best to add here that the pyrenoid CCM in Chlammy is inducible under conditions of low inorganic carbon present in the surrounding liquid environment.
- We thank the Reviewer for their kind comments and this suggestion. This sentence has now been amended to include 'under conditions of low inorganic carbon'.

2) Line 17 – The authors should define the acronym EPYC1 here and cite the original PNAS paper for its finding.
- EPYC1 has now been defined in the opening paragraph (now Line 18). The PNAS article by Mackinder et al. is already cited (6).

3) Lines 54-56: The authors mention a truncated form. I think chimeric form is better terminology. Or just the mature peptide of EPYC1. However, it is more like the mature EPYC1 peptide fused to the Arabidopsis RbcS1A transit peptide is required for proper chloroplast import within Arabidopsis and the subsequent removal of the TP by the stromal peptidase. Is there evidence from recent work by the authors that cleavage of the native EPYC1 TP from the full length EPYC1 peptide was occurring plants? Or is it a target of plant proteases because of incorrect processing or failure to assemble with Rubisco? Can the authors determine if the RbcS1A TP is correctly cleaved?
- In agreement with Reviewer 1, we have removed 'truncated' and replaced with 'mature'. Based on previous work (Atkinson *et al*., 2019 J Ex Bot), expression of the full EPYC1 produced a multiple banding pattern on immunoblots, which suggested that the presence of the native EPYC1 transit peptide led to proteolytic degradation, likely by plant proteases. In the present study, removal of the predicted EPYC1 transit peptide prior to fusion with the RbcS1A transit peptide resulted in a single band for EPYC1 when expressed *in planta* in the $S2_{Cr}$ background. The size of the band corresponded to mature EPYC1 with the RbcS1A transit peptide removed (i.e. 55.6 kDa for EPYC1::tGFP and 63.9 6kDa for EPYC1::eGFP, these values are given in the Figure 1 legend).

4) The authors prudently used two different versions of GFP fused to EPYC1. It appears that tGFP is responsible for elevated expression or the promoter is more efficient. The promoters should be defined in the legend in Figure 1 and also in the methods. Secondly, is the codon usage identical for both GFP coding sequences in each fusion? It is quite interesting as to why EPYC fused to tGFP is more expressed and/or assembles more readily with Rubisco. Is the mRNA for the EPYC::tGFP construct more abundant? The lack of degradation products implies that EPYC1, which is structurally disordered, is protected from proteases once assembled with Rubisco.
- The promoters used have now been defined in the methods section (line 200): 'The 35S cauliflower mosaic virus (CaMV) promoter and CsVMV (cassava vein mosaic virus) promoter were used to drive expression.', and similarly in the legend of Figure 1 with the addition (line 478): 'and driven by the 35S CaMV promoter (35S prom) or CsVMV promoter (CsVMV prom), respectively.'

We agree that the higher levels of expression for EPYC::tGFP was interesting. When comparing eGFP to tGFP using online tools (www.biologicscorp.com/tools/CodonUsageCalculator), we found that the codon usage for tGFP was better optimised for plants compared to eGFP. Furthermore, tGFP includes an intron, which is common strategy known to enhance heterologous gene transcript translation (e.g. Laxa, 2017 Front Plant Sci). Together, this suggested that tGFP might be translated more efficiently than eGFP. To investigate further (and address the Reviewer's enquiry), we performed qPCR analysis of EPYC1 expression on available plant material from the three Ep lines shown in Figure 1b (now included as Extended Data Figure 2). We found that the overall expression levels of EPYC1 transcripts were consistent with protein levels in Ep1-3, and similarly that eGFP was not expressed in Ep3 (we are not sure why). However, the transcript abundances of tGFP and eGFP were not significantly different for Ep1 and Ep2, indicating that tGFP translation is likely more efficient. On line 67 we have now replaced 'more highly expressed' with 'more abundant at the protein level (Fig. 1b, Extended Data Fig. 2)'.

5) The authors haven't cited the review by Wunder et al 2019 – Traffic. This is relevant to the study and describes appropriate background to EPYC1 and LLPS.
- This reference has now been added (22) and referred to on line 55.

6) The authors mention that the size of the condensate is likely related to expression level of EPYC1 but there is still degradation intermediates. Presumably, the free Rubisco could form another condensate within the chloroplast or is there some molecular crowding influence as well that

governs this process? The authors do provide evidence it's not chloroplast size which further suggests the microenvironment is somehow limiting the assembly.

- We do not know currently what limits the size of the condensate. In our system we have observed that condensate size corresponded to the expression level of EPYC1 (i.e. Figure 1b and Extended figure 3c), but did not exceed 2.4 µm in diameter. Similarly, in Chlamydomonas the maximum pyrenoid diameter observed was 2.2 µm. This differs from *in vitro* Rubisco-EPYC1 condensates, which are not size limited (Figure 3c and Wunder et al., 2018 Nat Comm). The ripening of liquid-liquid phase separated (LLPS) droplets can be determined by the stiffness of the surrounding network, which may explain why in the stroma (which is densely packed with thylakoids) the condensate appears size-limited. We have not observed a second condensate in the same chloroplast, which is likely due to the effects of Ostwald ripening, where surface tension leads to the growth of one single condensate, or elastic ripening, where the preferential growth of droplets at the region of least network stiffness may cause coalescence at a single point. A sentence in the manuscript has been extended to describe this (line 87-89), '…likely limited by the influence of the surrounding network stiffness (i.e. the stroma, which is densely packed with thylakoids) on the dynamics of droplet ripening[24].' , and a new reference (24) has been included: Rosowski, K. A et al. Elastic ripening and inhibition of liquid-liquid phase separation. Nature Physics 16, 422-425 (2020).

7) The authors mention that 52% of Arabidopsis Rubisco is incorporated into the proto-pyrenoid. This make sense since figure 3b shows a 1:1 ratio of Cr SSu's to Arabidopsis SSu's. However in Line 125, do the authors mean that in the condensate 81% of the SSu's are from Chlammy? This sentence is not clear.

- We apologise for any confusion. To clarify, the $S2_{Cr}$ background has both native Rubisco and hybrid Rubisco containing Chlamydomonas SSUs. Densitometry analyses of Coomasie gels indicated that nearly half (49%) of the Rubisco pool in $S2_{Cr}$ plants contains Chlamydomonas SSUs (line 125), which is consistent with measurements in Atkinson *et al*. 2017 (New Phytol). Our immunogold analyses of the condensate showed that 81% of all Chlamydomonas SSUs are located in the condensate, while a separate antibody showed that 54% of all Rubisco was in the condensate. Unfortunately, we were not able to determine from the TEM what proportion of Rubisco in the condensate contained Chlamydomonas SSUs, as the antibodies likely have different binding efficiencies. However, we did show that 82% of Rubisco in the condensate contained Chlamydomonas SSUs from densitometry of the Coomassie stained gel (line 125-126). We have now rephrased he sentences in lines 128-132 to improve clarity: 'Immunogold analysis of chloroplast TEM images from $S2_{Cr}$ plants expressing EPYC1-dGFP showed approximately half (54%) of all Rubisco was contained within the condensate (Fig. 3d, 3e, Extended Data Fig. 4b). Consistent with Coomasie staining, 81% of Rubisco containing Chlamydomonas SSU was located in the condensate.'

From figure 3b it appears that there is no Arabidopsis SSu's assembled in the condensate. However, the western blot shows perhaps a small amount. Perhaps a longer exposure maybe required for the western blot to accurately determine this. However, if there are no Arabidopsis SSu's present in the condensate, this also suggests that the SSu's are not assembled as mixed populations and points toward assembly is limited to one SSu population only. This needs to be further clarified.

- A band for the Arabidopsis AtRbcS1B/2B/3B isoforms (please note, subunit 3B is reduced, not absent, in the *1a3b* background band) is visible in both the immunoblot (Figure 3a) and the Coomassie stain (Figure 3b). We apologise that the resolution and/or contrast was not sufficient to see this and have adjusted both Figure 3a and 3b to improve the visibility of these bands. We have also renamed the Arabidopsis SSU band in the Coomasie stain to AtRbcS1B/2B/3B for clarity. The AtRbcS1A band is absent in the $S2_{Cr}$ background (i.e. *1a3b* expressing a Chlamydomonas SSU) and present in the WT. We agree with the Reviewer's second comment that we should clarify our current understanding of Rubisco and the SSU populations in the condensate and have added the following to the text (lines 138-142): ' It is currently unclear if Rubisco can form a heterogenous L8S8 complex with different SSU isoforms, or if only a single SSU isoform is favoured during assembly (Valegård *et al.,* 2018 Acta Crystallogr). Thus, it remains unclear whether the Rubisco pool within the condensate was comprised of a mixture of homogeneous Rubisco complexes, or those containing both Arabidopsis and Chlamydomonas SSUs.'

8) The photosynthetic competency of transgenic plants are similar to azygous controls which suggests that CO2 can penetrate the proto-pyrenoid to Rubisco molecules close the central part of the condensate. This is further evidenced by similarities in Vcmax. Have the authors measured Rubisco activation status? This might be important because within the condensate Rubisco activase might not be able to penetrate to remove inhibitors from the active sites of Rubisco. Because CO2 is not elevated within the proto-pyrenoid (as there is no bicarbonate pumps), it is likely that a significant proportion of Rubisco is not activated or competent for catalysis.

- We appreciate this observation. As the Reviewer points out, the transgenic plants appeared photosynthetically equivalent to azygous plants under the conditions tested. Plants were grown under typical conditions for Arabidopsis and under Rubisco-limiting conditions (high light), and for the latter we found no evidence of Rubisco-limited growth. Furthermore, the Reviewer highlights that V_{cmax} values were not significantly different – plants with significant proportion of inactive Rubisco would be expected to show a difference in several of the photosynthetic parameters measured, including as V_{cmax}. Given that Rubisco activase is present in the Chlamydomonas pyrenoid (McKay *et al.*, 1991 Protoplasma) and no diffusional limitations are predicted for the movement of CO_2 or RuBP between the stroma and pyrenoid in Chlamydomonas (Kuken et al., 2018 eLife), we expect that this is also be the case for condensates in plants. It will be interesting to measure the extent of Rubisco activation in the proto-pyrenoid in future characterisation work (e.g. on a functional CO_2-concentrating mechanism in higher plants), but we feel this is not within the scope of the current study.

9) It is great to see that the authors have used the appropriate Arabidopsis kinetic parameters to model the ACi curves to determine Vcmax and other parameters. However, I am not sure if the method described by authors to determine mesophyll conductance is robust enough. Generally, it is accepted that the most accurate method for determining gm is online carbon isotope discrimination coupled to photosynthesis measurements using the Licor-6400.

- Thank you for this comment. Although gas exchange and/or fluorescence methods to measure measuring mesophyll (g_m) are well established and often used, we agree that carbon isotope discrimination is a more robust method and we did discuss this with experts in the field. It was recommended that we first investigate if there were discernible differences in growth or physiological parameters derived from gas exchange and fluorescence measurements (including g_m). As we observed no significant differences under the conditions tested we decided not to progress with more detailed g_m characterisations (i.e. online carbon isotope discrimination).

10) As the authors point out, alterations to the Rubisco LSu requires plastid transformation that is not available in key C3 monocot crops, however, work by Salesse-Smith et al., 2018 Nature Plants, strongly suggests that there is an ability to express Rubisco LSu from the nucleus in maize plants. The authors should be aware of this work.

- We thank the Reviewer for this reminder and recommendation. Salesse-Smith et al. 2018 has now been cited (31) and the following sentence expanded (line 184): '…engineering of the chloroplast-encoded Rubisco large subunit, which is not generally feasible in major grain crops such as wheat and rice, although expression of the large subunit from the nucleus of maize plants has been recently demonstrated[31].'

11) Lines 149-151 – The authors suggest here that decreases in Rubisco content is the cause of differences in CO2 assimilation between Transgenic plants and WT. The authors have recently published the kinetic parameters of the hybrid Arabidopsis LSU / Chlammy SSu Rubisco which were significantly different from the WT Arabidopsis. The >10% reduction in the carboxylation efficiency of the hybrid Rubisco and decreases in Kcat and Sc/o is sufficient to induce changes in CO2 assimilation. A lower Rubisco content X a lower kcat reduces in vitro Vcmax significantly. I would suggest the authors reword this text to include reductions in Rubisco content and changes in Rubisco kinetics are responsible for the changes in CO2 assimilation.

- We agree and have included on line 152: ' …and differences in Rubisco catalytic characteristics'.

Reviewer #3 (Remarks to the Author):

The manuscript by Atkinson et al. reports on an approach to introduce the algal CO2 concentrating mechanism (CCM, a functional Rubisco condensate) into plant chloroplasts. In the chloroplasts of eukaryotic algae, Rubisco together with the linker protein EYPC1 localizes into a phase-separated compartment called pyrenoid. High concentrations of CO2 in the pyrenoid enhance the carboxylation function of Rubisco. Such a microcompartment is absent in C3 plants and the Rubisco operates under sub-optimal CO2 conditions. To increase crop yields, one important strategy would be to introduce an algal pyrenoid-like or cyanobacterial carboxysome-like Rubisco condensate into higher plant chloroplasts. Here the authors report the first successful attempt of forming a single phase separated compartment in Arabidopsis chloroplasts upon expression of EYPC1 and a plant-algal hybrid Rubisco. Although the Rubisco/EYPC1 condensate displaced the thylakoid membranes, the authors observed no impairment in Rubisco function and only a mild impairment in plant growth.
The approach reported by Atkinson et al. is promising, requiring modification of the nuclear-encoded SSU and possibly introducing a minimal set of genes to create an efficient algal CCM in plants. This strategy is possibly easier than introducing the CCM of cyanobacteria, which requires many more genes for carboxysome formation in plants.

Major points:
1. The authors used S2Cr instead of 1AAtMOD to carry out the whole study. 1AAtMOD has a better turnover rate and specificity comparing with S2Cr. Furthermore, as reported previously, the amount of total Rubisco is 75% of wild-type levels for 1AAtMOD, and 65% for S2Cr. Can the authors comment why they did not use the 1AAtMOD strain for this initial critical study of making a proto-pyrenoid in higher plant chloroplasts.
- We thank the Reviewer for their positive opening comments. The S2Cr background was chosen based on recent work showing the strength of interaction between EPYC1 and the Chlamydomonas SSU was significantly stronger than with the modified Arabidopsis SSU (1AAtMOD) (Atkinson et al., 2019 J Ex Bot). Furthermore, that work showed that condensation of EPYC1 and hybrid Rubisco from S2Cr plants could occur *in vitro*. As this was a proof-of-concept study focused on the feasibility of generating a proto-pyrenoid condensate in plant chloroplasts, we prioritised the S2Cr background. We also investigated expression of mature EPYC1 in 1AAtMOD plants (Figure 1c), but we found that it was a suboptimal model for condensate characterisation as the condensates were more diffuse (Figure 2a).

2. Lines 63-65: The statement that EPYC1::tGFP is generally more highly expressed than EPYC1::eGFP is not supported by the data shown in Figure 1b, the loading actin control for EPYC1::eGFP is much lower than that for EPYC1::tGFP.
- Lines 63-65 refer specifically to the expression of EPYC1::tGFP and EPYC1::eGFP from the dual GFP expression system (EPYC1-dGFP) in the Ep1-3 plants. In these plant lines EPYC1::tGFP is clearly more abundant than EPYC1::eGFP (Fig 1b). The right hand side of the blot in Fig 1b shows plant lines expressing either EPYC1::tGFP or EPYC1::eGFP. As the latter samples were meant to highlight the different sizes of these proteins rather than for quantitative comparison of expression, we feel that the protein loading in the immunoblot is not directly relevant.

3. Lines 92-93: Again the statement "However, the overall levels of EPYC1 expression in these lines were comparatively lower (Fig. 1b)." is not supported by the data shown in Figure 1b. In Fig. 1b, the EPYC1::eGFP or EPYC1::tGFP strains expressing "eGFP" and "tGFP" show higher levels of the respective proteins than in Ep1, Ep2 or Ep3.
- This sentence has now been removed (now line 97).

4. Description of the FRAP and chemical cross-linking experiments are missing in Methods section.
- We apologise for this omission, this has now been added (lines 280-289).

5. Please specify which transgenic Ep plant (1, 2 or 3) was used in the respective figures.
- We used plant line Ep3 - this information has now been added to the legends of Figure 1 (line 526), Figure 2 (line 537) and Figure 3 (line 551).

6. In Figs. 3a and 3b the amount of protein in the input is lower than the combined intensity of supernatant and pellet. Please clarify. The present figure is misleading.
- The volume of the resuspended pellet was small (20 µl) compared to the total volume of the input and supernatant (both *ca*. 800 µl). In order to show the presence of the native Arabidopsis SSU on the immunoblot, we chose not to further dilute the pellet sample. The following sentence has been added to Figure legend 3 (line 546) for clarity: 'The pellet is 40x more concentrated than the input and supernatant'.

7. It is surprising that essentially no AtRbcS was detected in the pelleted condensate (Figure 3b). It is hard to imagine that all eight RbcS binding sites on Rubisco are occupied exclusively by CrRbcS, considering that the total amount of AtRbcS protein appears to be slightly higher than CrRbcS2 (Figure 3b).
- A similar query was raised by Reviewer 2 (comment 7), and we have addressed this in detail there.

8. The values for Vmax and Jmax are not consistent with those reported previously in Atkinson, N. et al. (2017). Can the authors explain the difference?
- The parameters derived from our 2017 study were measured in plants grown under 25% lower light levels (i.e. 150 µmol photons m^{-2} s^{-1}). Furthermore, we have since started growing plants under LED lights rather than fluorescent bulbs we used previously. Even under equivalent light levels, we have observed that plants generally grow better under LED lights, which is why we have included details on the lights and growth cabinet used in the methods section. Together these environmental factors likely account for the differences in photosynthetic parameters observed.

Minor points:
1. Figure 1d; show also TEM of WT cells. Please indicate the "dense body" by an arrow (or arrowhead) and explain/describe what the white large structures are in the legend.
- A TEM image of EPYC1-dGFP expressed in a WT plant has now been included in Extended Data Figure 3. We believe that this provides a more useful control for comparison with EPYC1-dGFP expressed in the $S2_{Cr}$ background. No condensates were observed in WT expressing EPYC1-dGFP, and chloroplasts from WT plants appeared similar to the non-transformed $S2_{Cr}$ background (Fig 1d, left). The EPYC1-dGFP condensate is now indicated by an arrowhead. The legend now includes the following (line 516): 'A white arrowhead indicates the dense dark grey area of the EPYC1 condensate. The large white structures are starch granules. Scale bars = 0.5 µm. A representative chloroplast from a wild-type plant expressing EPYC1-dGFP is shown for comparison in Extended Data Figure 3.'

2. Figure 1f: why is Rubisco condensate not seen in all chloroplast organelles?
- The field of view is limited such that for some chloroplasts only one side is visible, and that side may not contain the Rubisco condensate.

3. Figure 1f: please describe the magenta puncta in the figure legend.
- We have added the following to the legend (line 522): 'The magenta puncta show the position of grana stacks.'

4. Figures 1f and 1g: What are the light pink/white structures in the condensate? Are these regions representing overlap of the condensate and thylakoid chlorophyll?
- Yes, the lighter magenta structures are thylakoid membranes behind or in front of the condensate. No thylakoid membranes appeared to enter inside the condensate (see also Extended Data Video 1). We have now included in the legend (line 522): 'Light magenta puncta indicate grana stacks behind or in front of the condensate.'

5. Please keep the images within the same figure panel in the same scale. This applies to Figure 1c, Extended Data Figure 2b and Extended Data Figure 3a.
- We have now adjusted the confocal images in Figure 1c, Extended Data Figure 3b (was 2b) and Extended Data Figure 4a (was 3a) to be at the same scale.

6. Figure 2a: A dotted line at y=1.0 would be helpful to indicate that the fluorescence signal has been normalized to 1.

- We have included a dashed line in Figure 2a and added to the legend (line 534): '(as indicated by the dashed line)'.

7. In Figure 2b the position of bleach point should have been in the middle of the condensate to better visualize recovery. The pre-bleach image of the fixed sample looks overexposed. Was the display setting for both samples the same? Was the fluorescence of GFP lower after fixing? Could this be due to denaturation of GFP?

- The aggregates were not sufficiently large enough to bleach a region in the centre and still have an equivalently sized un-bleached region at the sides for comparison. We found the assay was more consistent when bleaching a region towards one side of the condensate and comparing this to an equivalent non-bleached area. The imaging settings for both fixed and non-fixed samples were the same, but there were minor variations in how long it took to focus the laser for bleaching, such that the GFP signal in some condensates was lower to start compared to others. Thus, all data was normalised to the starting fluorescence. The brightness setting in Figure 2b was enhanced for greater clarity in our initial submission, which arguably gave the appearance of being over-exposed. We have now modified Figure 2b with non-enhanced images.

8. Lines 119 & 123, & Figure 3b: Correct spelling 'Coomassie'

- This has been corrected.

9. Figure 3d: In the image of CrRbcS2, what are the large dark circular clusters? The authors should indicate the chloroplasts with a dotted outline and indicate the Rubisco condensate. Again what are the large white structures?

- The large dark clusters are contamination from forceps used whilst handling the ultrathin sections. We do not feel that this impacts on the data as the clusters are visually very distinct from the gold nanoparticles used for immunogold labelling, and the latter have been highlighted with circles for clarity. We feel that the chloroplasts are clearly visible and the main subjects of the images, and that putting a dotted outline around the outside would detract from the images. The condensates have been labelled by arrowheads and the following has been added to the legend (line 553): 'The condensates are marked by a white arrowhead. Large white structures are starch granules.'.

10. Figure 3e is not described or mentioned in the text. Why is the y-axis scale set at a maximum of 1.5 when the maximum value of data is only 1.0?

- Thank you for highlighting this - Figure 3e has now been referenced in the text (Line 128). The y-axis for Figure 3e has been modified.

11. Extended Data Figure 3b: please indicate the chloroplasts with observable Rubisco condensates and also the scale bar is missing.

- Arrowheads have been added (now Extended Data Figure 4b) and highlighted in the legend (line 720): ' Visible condensates are marked by a white arrowhead.' A scale bar has also been added.

12. A better description of Az lines would be helpful for a non-expert.

- We have clarified in the sentence starting on line 145 '…azygous segregant $S2_{Cr}$ lines where the EPYC1-dGFP transgene was absent (Az1-3).'

13. Please site Figure 1c and the previous publication at the end of the statement (lines 153-156), "Condensate formation in the 1AAtMOD background, where catalytic characteristics of the hybrid Rubisco are indistinguishable from that of WT Rubisco, indicates that the SSU can be further engineered to optimise phase separation, and Rubisco content and performance."

- A citation has been included (now line 170).

14. Please site Figure 4d at the end of the statement (lines 145-147), "Notably, the CO2 assimilation rates at ambient concentrations of CO2 for Ep and Az lines were comparable to WT lines when

normalized for Rubisco content (ARubisco)”.
- A citation has been included (now line 161).

15. Extended Data Figure 4 legend, change “a)” and “b)” to “a,” and “b,”, respectively.
- This has now been changed.

Reviewer #1 (Remarks to the Author):

The revised manuscript has been significantly improved.

Reviewer #2 (Remarks to the Author):

The manuscript by Atkinson et al has been thoroughly revised in accordance with my comments. This manuscript represents a significant advance to the field and I have no further comments to make.

Reviewer #3 (Remarks to the Author):

I have no further comments.
